# Plasma Fatty Acid Binding Protein 4 and Risk of Sudden Cardiac Death in Older Adults

**DOI:** 10.1155/2013/181054

**Published:** 2013-12-28

**Authors:** Luc Djoussé, Marlena Maziarz, Mary L. Biggs, Joachim H. Ix, Susan J. Zieman, Jorge R. Kizer, Rozenn N. Lemaitre, Dariush Mozaffarian, Russell P. Tracy, Kenneth J. Mukamal, David S. Siscovick, Nona Sotoodehnia

**Affiliations:** ^1^Division of Aging, Department of Medicine, Brigham and Women's Hospital and Harvard Medical School, 1620 Tremont Street, 3rd Floor, Boston, MA 02120, USA; ^2^Boston Veterans Affairs Healthcare System, Boston, MA 02130, USA; ^3^Department of Biostatistics, University of Washington, Seattle, WA 98195, USA; ^4^Nephrology Section, Veterans Affairs San Diego Healthcare System, San Diego, CA 92161, USA; ^5^Divisions of Nephrology and Preventive Medicine, University of California San Diego, San Diego, CA 92093, USA; ^6^National Institute on Aging, National Institutes of Health, Bethesda, MD 20892, USA; ^7^Cardiology Division, Albert Einstein College of Medicine, New York, NY 10461, USA; ^8^Cardiovascular Health Research Unit, Departments of Medicine, University of Washington, Seattle, WA 98195, USA; ^9^Department of Medicine, Brigham and Women's Hospital and Harvard Medical School, Boston, MA 02120, USA; ^10^Department of Pathology and Biochemistry, University of Vermont College of Medicine, Burlington, VT 05401, USA; ^11^Division of General Medicine and Primary Care, Beth Israel Deaconess Medical Center and Harvard Medical School, Boston, MA 02120, USA; ^12^Cardiovascular Health Research Unit, Departments of Medicine and Epidemiology, University of Washington, Seattle, WA 98195, USA

## Abstract

Although fatty acid binding protein 4 (FABP4) may increase risk of diabetes and exert negative cardiac inotropy, it is unknown whether plasma concentrations of FABP4 are associated with incidence of sudden cardiac death (SCD). We prospectively analyzed data on 4,560 participants of the Cardiovascular Health Study. FABP4 was measured at baseline using ELISA, and SCD events were adjudicated through review of medical records. We used Cox proportional hazards to estimate effect measures. During a median followup of 11.8 years, 146 SCD cases occurred. In a multivariable model adjusting for demographic, lifestyle, and metabolic factors, relative risk of SCD associated with each higher standard deviation (SD) of plasma FABP4 was 1.15 (95% CI: 0.95–1.38), *P* = 0.15. In a secondary analysis stratified by prevalent diabetes status, FABP4 was associated with higher risk of SCD in nondiabetic participants, (RR per SD higher FABP4: 1.33 (95% CI: 1.07–1.65), *P* = 0.009) but not in diabetic participants (RR per SD higher FABP4: 0.88 (95% CI: 0.62–1.27), *P* = 0.50), *P* for diabetes-FABP4 interaction 0.049. In summary, a single measure of plasma FABP4 obtained later in life was not associated with the risk of SCD in older adults overall. Confirmation of our post-hoc results in nondiabetic people in other studies is warranted.

## 1. Introduction

Each year, nearly half a million sudden cardiac deaths (SCDs) occur in the US [1, 2]. Although more than 80% of SCDs occur in patients with coronary heart disease or congestive heart failure, conditions strongly linked to adiposity [3–5], several traditional adiposity-related risk factors such as high cholesterol or high blood pressure are not strongly predictive of SCD. Adiposity [6] and metabolic syndrome [7] have been positively associated with an increased QT dispersion, another risk factor for SCD [8–10]. Furthermore, weight loss may lead to a decrease in QT dispersion [11], suggesting that adiposity may influence the risk of SCD.

Adipose tissues produce various adipokines including fatty acid binding protein 4 (FABP4)—also referred to as aP2 or a FABP, a carrier protein that transports fatty acids and other lipophilic substances between extra- and intracellular membranes [12–14] and exerts diverse effects on modulation of inflammation, thrombogenicity, insulin resistance, and other metabolic pathways [15–18]. In isolated rat cardiomyocytes, FABP4 acutely depressed shortening amplitude and intracellular systolic peak Ca(2+) in a dose-response fashion [19]. This suggests that FABP4 may play an important role in cardiac depolarization and possibly cardiac arrhythmias. However, to our knowledge, no data are available in humans on the effects of FABP4 on cardiac arrhythmia. Because FABP4 may also increase the risk of type 2 diabetes, it is possible that people with diabetes may be at a higher risk of SCD than nondiabetic individuals. Furthermore, while limited and inconsistent data have been reported on the association between body mass index and SCD among heart failure subjects (no association) [20] or postmyocardial infarction (41% increased risk of SCD per 5 unit decrease in BMI) [21] patients, no previous study has examined whether FABP4 influences the risk of SCD in a community setting. Understanding the role of FABP4 on SCD is important as FABP4 could potentially serve as a novel pharmacological target in the prevention of SCD. Therefore, we sought to examine the association between plasma FABP4 and incident SCD.

## 2. Methods

### 2.1. Study Population

The Cardiovascular Health Study (CHS) is a prospective, population-based cohort study of cardiovascular disease in older adults. In 1989-1990, 5201 men and women aged 65 years were recruited from a random sample of Medicare-eligible residents in the following 4 US communities: Forsyth County, NC; Sacramento County, CA; Washington County, MD; and Allegheny County, PA. A supplemental cohort of 687 predominantly African-Americans was recruited in 1992-1993 from three of the same communities (excepting Washington County) by using the same sampling and recruitment methods. The institutional review board of each center approved the study, and all participants gave written informed consent to participate in the study. Details of the study design, sampling, and recruitment have been published [22]. The 1992-93 examination served as the baseline for this analysis. Of the 4,707 participants for whom FABP4 measurements were available, we excluded 147 who were missing covariates included in the main analysis (final sample of 4560 subjects for current analyses). In a secondary analysis involving prevalent diabetes, an additional 77 participants were excluded due to missing diabetes status at the study baseline, reducing the sample to 4483 individuals.

### 2.2. Measurement of Plasma FABP4

Plasma samples collected at the 1992-1993 examination were stored at −70°C until analyzed at the central laboratory at the University of Vermont. Plasma FABP4 concentration was measured using standard ELISA kits (BioVendor ELISA). The inter-assay coefficient of variation was 2.61% to 5.32% (detectable range 5 to 250 ng/mL).

### 2.3. Assessment of SCD in the Cardiovascular Health Study

Details on ascertainment and classification of cardiovascular events and deaths in CHS have been published previously [23, 24]. Cause of death was adjudicated by a centralized CHS Events Subcommittee by review of death certificates, inpatient medical records, nursing home or hospice records, physician questionnaires, interviews with next-of-kin, and autopsy records, where available. SCD was defined as a sudden pulseless condition, presumed due to cardiac arrhythmia, in a previously stable individual that occurred out of the hospital or in the emergency room. For unwitnessed deaths, the participant must have been seen within 24 hours of the arrest in a stable condition and without evidence of a noncardiac cause of cardiac arrest. By definition, SCD cases could not be under hospice or nursing home care or have a life-threatening noncardiac comorbidity. Medical records for all cardiac deaths adjudicated by the CHS Events Subcommittee were further reviewed by a cardiologist in order to identify and classify SCD cases. A blinded second physician review of a random sample of 70 of these death records showed an 88% inter-reviewer agreement and *κ* = 0.74 for SCD. SCD was classified as definite, probable, or possible, depending on the level of evidence available. Initial analyses were restricted to definite or probable SCDs. The present analysis included SCDs occurring through June 2006, the latest date of adjudicated SCD events.

### 2.4. Other Variables

Aside from age, race and education which were ascertained in 1989-90 for the participants in the first enrollment wave, all other covariates used in the analysis were based on data obtained during the 1992-1993. Information on age, race, years of education, smoking status, hormone replacement therapy, general health status, hypertensive medication use, lipid lowering medication use, and alcohol consumption was based on self-report. Leisure-time activity (kcal/week) was assessed using a modified Minnesota Leisure-Time Activities questionnaire [25]. Weight, height, and waist circumference were measured using standardized protocols by trained study personnel. Body mass index (BMI) was calculated as weight in kilograms divided by height in meters squared. Total cholesterol was measured in fasting blood samples by standard assays. High-sensitive C-reactive protein (hsCRP) was measured by standard ELISA, and glomerular filtration rate was calculated based on cystatin measurements as previously described [26].

### 2.5. Statistical Methods

We fit Cox proportional hazards models to estimate the relative risk (RR) of SCD associated with an increase of one standard deviation in FABP4 levels (SD = 18.98 ng/mL). Time at risk was calculated as the interval in days from the date of the 1992-93 examination to the earliest of date of SCD, date of death from another cause, or the end of SCD followup in 2006. An initial model adjusted for age, sex, race, and field center (Model 1). A second model additionally adjusted for education (high school versus less), GFR-cystatin, CRP, physical activity, alcohol consumption (none, 7 or less drinks per week, more than 7 per week), smoking (never, former, current), health status (poor, fair, good, very good, excellent), and hormone replacement therapy (Model 2). In Model 3, we also adjusted for body mass index.

Because FABP4 may increase the risk of diabetes and women have higher plasma levels than men, we examined in secondary analyses FABP4-by-diabetes and FABP4-by-sex interactions using product terms.

As a sensitivity analysis, we additionally adjusted for waist circumference in Models 1–3. Given the influence of weight loss on plasma concentration of FABP4 [27, 28], we repeated our main analyses and the analysis adjusting for waist circumference after exclusion of individuals with cancer, cardiovascular disease, or reported unintentional weight loss in the past 12 months (*n* = 1821). Unintentional weight loss was defined as a loss of more than 10 pounds not due to exercise or diet. Subjects who were missing information on weight loss, diet, or exercise were not considered as subjects that experienced unintentional weight loss. Lastly, we additionally controlled for self-reported unintentional weight change in the multivariable analysis.

To evaluate the effect of coronary heart disease (CHD), congestive heart failure (CHF), hypertension, and hypercholesterolemia as potential intermediate factors between FABP4 and SCD, we fit a model with additional adjustment for CHD, CHF as well as systolic blood pressure, hypertension medication use, and hypercholesterolemia. Hypercholesterolemia was defined as the use of lipid lowering drugs or total cholesterol > 200 mg/dL. We evaluated the validity of the proportional hazards assumption by using Schoenfeld residuals and found no meaningful departures. Statistical analysis was performed in R 2.13.0 (http://www.r-project.org/).

## 3. Results

Among 4,560 participants, 2,648 were women (58.1%), and the mean age at baseline was 74.9 years (range 65 to 98 years). Median plasma FABP4 was 29.97 ng/mL (IQR 22.41−40.76), and women had substantially higher concentrations of FABP4 (median 35.35 ng/mL) than men (median 23.62 ng/mL, *P* ≤ 0.001.

Table 1 presents baseline characteristics of participants according to tertiles of FABP4. Compared with the lowest tertile of FABP4, those in the highest tertile had higher measures of adiposity, *P* ≤ 0.001, and were more likely to be women, *P* ≤ 0.001, or African-American, *P* ≤ 0.001; persons in the highest tertile of FABP4 were more likely to be physically inactive, *P* ≤ 0.001, had higher hsCRP, *P* ≤ 0.001, and had lower prevalence of current smoking, *P* ≤ 0.001, alcohol consumption, *P* ≤ 0.001, and self-reported poor health status, *P* ≤ 0.001. During a median followup of 11.8 years, 146 cases of “definite” or “probable” SCD were documented. The crude incidence rate of SCD was 3.1, 2.9, and 3.7 cases per 1000 person-years across consecutive tertiles of FABP4. In a Cox regression model adjusting for age, sex, and field center, each SD of FABP4 was associated with a 32% higher risk of SCD (95% CI: 16% to 51%), *P* ≤ 0.001 (Table 2). However, upon additional adjustment for education, glomerular filtration rate based on cystatin, hsCRP, leisure time physical activity, hormone replacement therapy, alcohol intake, self-reported general health status, smoking, and body mass index, this association was no longer statistically significant (HR: 1.15 (95% CI: 0.95–1.38)), *P* = 0.15 (Table 2). Adding potential intermediate factors (congestive heart failure, coronary heart disease, systolic blood pressure, and use of antihypertensive or cholesterol-lowering drugs yielded an HR of 1.12 (0.92–1.37), *P* = 0.24. When we repeated the main analysis after including an additional 74 SCD cases classified as “possible”, we observed similar results (RR per standard deviation increase of FABP4 was 1.12 (95% CI: 0.94–1.32), *P* = 0.20 in model 3).

When covariates were examined one at a time, glomerular filtration rate based on cystatin C was the single important confounder, attenuating the relative risk from 1.32 (95% CI: 1.16–1.51), *P* ≤ 0.001, to 1.18 (95% CI: 1.00–1.40), *P* = 0.055 (Table 3). Exclusion of 1821 participants with reported unintentional weight loss over the past year, cancer, and cardiovascular disease had little effect on the results (RR per standard deviation higher FABP4 1.18 (95% CI: 0.82–1.69), *P* = 0.35 in model 3).

In secondary analyses stratified by prevalent diabetes, FABP4 was associated with a higher incidence rate of SCD in nondiabetic participants, (HR per SD of FABP4: 1.33 (95% CI: 1.07–1.65), *P* = 0.009) but not in diabetic participants (HR per SD of FABP4: 0.88 (95% CI: 0.62–1.27), *P* = 0.50), *P* for diabetes-FABP4 interaction 0.049 (Table 4). There was no evidence for interaction by sex (*P* for sex-FABP4 interaction is 0.20, 0.33, 0.33 for models 1–3, resp.), and the positive association between FABP4 and SCD among nondiabetic subjects was present in both men (HR: 1.75 (1.35–2.28), *P* < 0.001) and women (HR: 1.39 (1.14–1.69), *P* = 0.001) (analysis adjusted for age, race, and clinic). There was no interaction between BMI and FABP4 on the risk of SCD (*P* = 0.32). Lastly, to explore possible effects of weight change on the FABP4-SCD relation, we repeated the main analysis using people with unmissing data on weight change and observed HR of 1.05 (95% CI: 0.84–1.31) using model 3 without versus HR of 1.04 (95% CI: 0.83–1.30), *P* = 0.72, with additional adjustment for weight change.

## 4. Discussion

In this large prospective study among community-dwelling US older adults, we observed no association between FABP4 and the risk of SCD. In a post-hoc analysis stratified by prevalent diabetes, we demonstrated a higher risk of SCD per SD higher FABP4 in nondiabetics but not in people with diabetes (*P* for interaction 0.049). To the best of our knowledge, this is the first study to evaluate the association between FABP4 and SCD.

Despite a lack of direct evidence of an association between FABP4 and SCD in humans, previous studies have reported associations between FABP4 and risk factors for SCD, including coronary artery disease. Specifically, Terra et al. [29] reported a positive association between FABP4 and C-reactive protein, tumor necrosis alpha receptors, and interleukin-6 in a sample of 81 morbidly obese females. Furthermore, FABP4 has been inversely related to adiponectin and positively related to adiposity [17], metabolic syndrome [17, 18, 30], coronary heart disease burden [31–34], and diabetes [35].

Chronic kidney failure is a major risk factor for coronary disease [36, 37] and people with impaired kidney function have been reported to have higher concentrations of FABP4 [38, 39]. In addition, FABP4 has been shown to correlate with oxidized LDL and poor endothelial function in diabetic subjects [40]. Oxidized LDL has been reported to induce the expression of FABP4 mRNA in human macrophages [41]. These data suggest that FABP4 may indirectly influence the risk of SCD via incident coronary disease. On the other hand, both adiposity [6] and metabolic syndrome [7] have been positively associated with an increased QT dispersion, another risk factor for SCD [8–10]. Furthermore, weight loss has been shown to decrease QT dispersion [11]. In our study, exclusion of 236 subjects with unintentional weight loss led to a slight increased estimate of association (RR per SD higher FABP4 was 1.15 (95% CI: 0.95, 1.38) before and 1.20 (95% CI: 0.99, 1.47) after such exclusion). This slight change suggests that people with other comorbidities related to a higher SCD risk may be prone to experience weight loss and end up in the reference group, thereby diluting the effect measure. Our post-hoc results in nondiabetic warrant future confirmation in other cohorts.

Limited data available in this area are from *ex-vivo* experiments. For example, in isolated rat cardiomyocytes, FABP4 was shown to acutely depress shortening amplitude and intracellular systolic peak Ca(2+) in a step-wise fashion [19]. This suggests that FABP4 may play an important role in cardiac depolarization and possibly cardiac arrhythmias. FABP4 has been positively associated with conditions that could increase the risk of SCD including coronary artery disease [32, 34], metabolic syndrome [17, 42], overall insulin resistance [43–45], and incident diabetes [35, 46]. Our group has reported a positive association between FABP4 and incident heart failure in this cohort [47]. However, our main analyses do not lend support to the hypothesis that FABP4 is a major risk factor for SCD in older adults.

The current study has some limitations. We only had a single measure of plasma FABP4 obtained after the age of 65 years in this cohort. It is unclear whether we would have obtained similar results with earlier measures of FABP4 (at younger age). Furthermore, we were not able to account for change in this biomarker over time, especially those due to weight change and other factors over time. Weight loss is associated with a reduction in serum FABP4 in humans [27, 28] and in our study, subjects in the highest tertile of FABP4 were more likely to report unintentional weight loss. Given the observational nature of our study, we are not able to exclude residual or unmeasured confounding as alternative explanation of the observed relation. Participants in our sample were predominantly Caucasian aged 75 years on average. Hence, our results may not be generalizable to younger individuals or other ethnic groups. Despite these limitations, our study has numerous strengths including a large sample size; a representative sample of older adults; inclusion of both men and women; the use of a valid and reproducible method to assess FABP4; a validation of SCD by cardiologists; availability of data on numerous potential confounders; and long-term and nearly complete followup.

## 5. Conclusions

In conclusion, a single measure of plasma FABP4 obtained later in life was not associated with the risk of SCD in older adults.

## Figures and Tables

**Table 1:** Baseline characteristics of the 4,560 participants according to tertiles of FABP4.

	Tertiles of FABP4 (ng/mL)			
	T1 (low)	T2	T3 (high)	
*N*	1,520	1,520	1,520	
Mean ± SD	19.4 ± 3.7	30.2 ± 3.2	53.3 ± 21.4	
Range	[5.8–24.9]	[25.0–36.4]	[36.5–250.0]	
Characteristics				*P* value
Age (years)	74.8 ± 5.0	74.7 ± 5.3	75.0 ± 5.5	0.29
BMI (kg/m^2^)	24.8 ± 3.6	26.6 ± 3.9	29.3 ± 5.5	<0.001
Waist circumference (cm)	93.0 ± 11.0	97.1 ± 11.9	102.8 ± 14.7	<0.001
Male (%)	70.1	37.7	17.9	<0.001
African American (%)	13.8	16.3	20.2	<0.001
Physical activity (kcal/week)^†^	1155 [490–2283]	810 [270–1907]	613 [140–1500]	<0.001
Smoking (%)				
Never	37.3	46.6	52.1	<0.001
Former	51.3	43.8	39.1	
Current	11.4	9.6	8.9	
Alcohol consumption (%)				
None	46.5	54.2	63.8	<0.001
≤7 drinks per week	38.4	35.4	30.1	
>7 drinks per week	15.1	10.4	6.1	
Health status (%)				
Poor	8.5	6.9	4.4	<0.001
Fair	36.0	31.0	26.2	
Good	38.8	43.1	42.7	
Very good	15.1	17.2	23.2	
Excellent	1.6	1.7	3.4	
Less than high school (%)	75.4	74.0	70.0	0.001
Unintentional weight loss (%)	4.9	4.1	6.5	0.05
** **GFR-cystatin C (mL/min/1.73 m^2^)	79.4 ± 17.3	73.8 ± 17.3	64.0 ± 18.5	<0.001
hsCRP (mg/L)^†^	1.8 [0.87–4.1]	2.6 [1.3–5.4]	4.2 [1.9–8.8]	<0.001
Prevalent coronary disease (%)	21.9	20.1	22.7	0.60
Prevalent heart failure (%)	4.0	5.0	8.9	<0.001
Estrogen use—women (%)	19.6	15.1	10.2	0.01
Hypertension medication (%)	40.4	48.8	63.2	<0.001
Systolic BP	135.0 ± 21.0	136.1 ± 21.7	137.8 ± 21.8	<0.001
Diastolic BP	71.5 ± 11.4	71.8 ± 10.9	70.6 ± 11.8	0.02
Total cholesterol (mg/dL)	192.1 ± 36.1	204.5 ± 36.6	208.8 ± 41.5	<0.001
Lipid lowering medication (%)	5.5	8.5	8.5	0.002
Hypercholesterolemia (%)	41.6	56.1	59.7	<0.001

Data are presented as mean ± SD or percentage, unless specified otherwise; hsCRP: high-sensitive C-reactive protein; GFR: glomerular filtration rate based on cystatin C.

^†^Median (interquartile range).

**Table 2:** Hazard ratio (95% CI) of SCD per each SD (18.98 ng/mL) increase of FABP4 concentration among CHS participants (*n* = 4560).

	Hazard ratio (95% CI)	*P* value
Per one SD higher FABP4		
Model 1*	1.32 (1.16–1.51)	<0.001
Model 2**	1.15 (0.96–1.37)	0.13
Model 3***	1.15 (0.95–1.38)	0.15

*Model 1: adjusted for age, sex, race, and field center.

**Model 2: adjusted for variables in model 1 plus education, GFR-cystatin, hsCRP (log transformed), kcal of leisure time physical activity (log transformed), hormone replacement therapy, alcohol, self-reported health status, and smoking.

***Model 3: adjusted for variables in model 2 plus body mass index.

**Table 3:** Effect of each covariate added one at a time on the hazard ratio (95% CI) of SCD per standard deviation (18.98 ng/mL) increase in FABP4.

Model	HR (95% CI)	*P* value
Model 1 (basic model)*	1.32 (1.16–1.51)	<0.001
Added covariates		
Less than high school	1.32 (1.16–1.51)	<0.001
GFR-cystatin	1.18 (1.00–1.40)	0.055
Serum creatinine	1.35 (1.16–1.57)	<0.001
hsCRP	1.27 (1.11–1.46)	<0.001
Kcal of leisure time activity (log)	1.32 (1.16–1.51)	<0.001
Alcohol consumption	1.32 (1.16–1.50)	<0.001
Self-reported health status	1.29 (1.13–1.48)	<0.001
Smoking	1.32 (1.17–1.51)	<0.001
Estrogen use (women)	1.32 (1.16–1.50)	<0.001
Body mass index	1.32 (1.15–1.51)	<0.001
Waist circumference	1.30 (1.13–1.50)	<0.001
Hypercholesterolemia	1.32 (1.16–1.51)	<0.001
Systolic blood pressure	1.32 (1.16–1.50)	<0.001
Hypertensive mediation use	1.26 (1.09–1.45)	0.001
Cardiovascular heard disease	1.29 (1.13–1.47)	<0.001
Cardiovascular heart failure	1.24 (1.08–1.44)	0.003
Diabetes	1.27 (1.11–1.45)	<0.001

*Model 1 adjusted for age, sex, race, and field center.

**Table 4:** Hazard ratio (95% CI) of SCD per each SD (18.98 ng/mL) increase of FABP4 concentration, by diabetes status (*n* = 4483).

	Diabetics (*n* = 682)		Nondiabetics (*n* = 3801)		
	HR (95% CI)	*P* value	HR (95% CI)	*P* value	*P* interaction^†^
Model 1*	0.95 (0.70–1.28)	0.72	1.45 (1.26–1.67)	<0.0001	0.056
Model 2**	0.81 (0.56–1.17)	0.26	1.35 (1.10–1.65)	0.004	0.050
Model 3***	0.88 (0.62–1.27)	0.50	1.33 (1.07–1.65)	0.009	0.049

*Model 1: adjusted for age, sex, race, and field center.

**Model 2: adjusted for variables in model 1 plus GFR-cystatin, hsCRP (log transformed), kcal of leisure time physical activity (log transformed), hormone replacement therapy, alcohol, health status, and smoking.

**Model 3: adjusted for variables in model 2 plus body mass index.

^†^Interaction between diabetes status and FABP4.

There were 41 SCDs in diabetic participants and 102 SCDs events in non-diabetic participants.
